# Allelism of Uncharacterized Dwarf Mutants in Maize

**DOI:** 10.17912/micropub.biology.001504

**Published:** 2025-02-07

**Authors:** Jeffery L Gustin, Shane A Zimmerman, Martin M Sachs

**Affiliations:** 1 USDA-ARS, Maize Genetics Cooperation Stock Center, Urbana, Illinois

## Abstract

Gibberellic acid (GA) is a phytohormone that is important for plant growth and development. Mutants in GA biosynthesis, signaling and metabolism have been critical to understanding the role GA plays in plants. GA mutants have also revolutionized global production of staple crops such as rice, wheat, and barley. GA mutants have been isolated in maize and characterization of the underlying genes has helped map the GA biosynthesis and signaling pathways. However, the number of maize dwarf mutants is far less than other species. Here, we identify new dwarf mutants that could benefit our understanding of maize plant height control.

**Figure 1. Allele test results between d* stocks and an1 , d1 , d3 , and d5 mutants:**
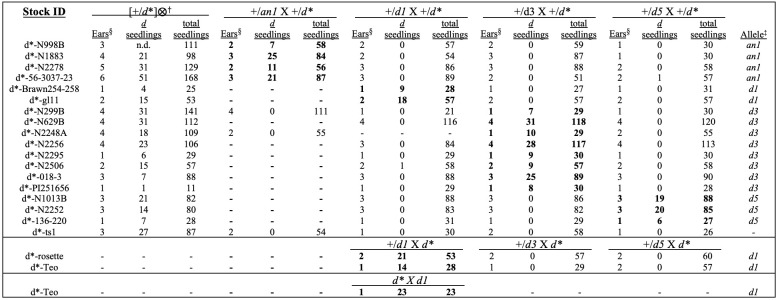
^§^ Number of segregating ears used in the allele tests. ‡ Probable allele of the *d* * stock. ^†^ The ⨂ symbol indicates a self-pollination. Positive allele test results are bolded.

## Description

Genetic mutants in the GA synthesis, signaling and metabolism pathways have been essential in identifying genes involved in the many roles that GAs play in plants (Reid, 1986, Ross et al., 1997; Yamaguchi, 2008; Hedden and Thomas, 2012) . Novel mutants continue to be identified and continue to expand our understanding of GA action, particularly in maize where the characteristic phenotypic effects of GA deficiency have made GA mutants readily identifiable. There is renewed interest in incorporating dwarfing genetics into commercial maize germplasm. However, identification and characterization of dwarf and semi-dwarf mutants in maize lags far behind that of other crops such as rice (Asano et al., 2009) .

Severe deficiency in GA biosynthesis or signaling in maize plants manifests a characteristic phenotype. Mutants fail to elongate internodes causing a rosette type (dwarf) plant architecture that also displays broad leaves. Tassels tend to appear thick and fail to extrude anthers due to poor filament elongation and ears display perfect, rather than female, florets (andromonoecious) (Phinney, 1956; Reid, 1986; Dellaporta and Calderon-Urrea, 1994) . The phenotype is readily observable in the field and mutagenesis projects have identified numerous rosette dwarf mutants. Cloning and characterization of the mutated genes that cause the severe dwarf phenotype has found that they are primarily involved in GA biosynthesis or GA signal transduction.

Seven enzymes are required to synthesize bioactive forms of GA (GA _1_ , GA _3_ and GA _4_ ) (reviewed by Yamaguchi, 2008; Hedden and Thomas, 2012) and maize mutants have been identified in 4 of the 7 steps. The enzymes encoded by genes *dwarf1 (d1)* , *d3,* *d5, and anther ear1 (an1)* all have roles in biosynthesis, and mutations of any one of them produce the classic rosette phenotype. The hydroxylation step catalyzed by GA20Ox enzyme is particularly noteworthy. Recessive dwarf mutants have not been identified for this enzymatic step most likely due to multiple genes encoding GA20ox enzyme activity. However, Paciorek et al., 2022 demonstrated that reducing the expression of two of the eleven genes encoding GA20Ox’s substantially reduced levels of bioactive GA in elongating tissues and reduced plant height by 1/3 ^rd^ with no significant effects on inflorescence tissues. The resulting short stature corn significantly increased harvest index and could positively impact on commercial maize production (Kosola et al., 2023) .

The GA signal transduction pathway is mediated by DELLA proteins, which are negative regulators of GA signaling. In the presence of bioactive GA, DELLA proteins are targeted for degradation, thereby triggering a GA response. Mutations in the highly conserved N-terminal region of the DELLA protein can stabilize the protein by preventing GA-induced degradation. This prevents GA signal transduction resulting in a dominant dwarf phenotype. Dominant DELLA mutations have been identified in many species and have made important contributions to increased crop production in wheat (‘green revolution’ gene Reduced height (Rht1d), rice (Slender rice1) and barley (Slender1) (Peng et al. 1999; Ikeda et al. 2001; Spielmeyer et al. 2002) . In maize, D8 (Zm00001eb054480) and D9 (Zm00001eb216610) encode DELLA proteins and dominant mutants *D8* and *D9* show a severe rosette dwarf phenotype similar to recessive dwarf mutants, *d1* , *d3* , and *d5* . Interestingly, mutations in the C-terminal region of the rice DELLA protein eliminate DELLA repression of GA signaling and causing a hypersensitive GA response resulting in tall, slender rice.

Bioactive GA levels are also regulated by several deactivation pathways. GA2ox enzymes add a 2b hydroxl group to bioactive GA’s (and inactive precursors), which sequesters these molecules into an inactive state (Thomas et al, 1999) . Deactivation can also occur via conjugation to molecules such as glucose (Von Schirach–Szmigiel, 1979; Zeng et al., 2024) To our knowledge, no maize mutants have been identified that impact GA deactivation despite numerous mutants in *Arabidopsis* and rice.

The Maize Genetics Cooperation Stock Center manages a large collection of maize mutants collected from maize geneticist from around the world. The collection includes mutants whose molecular lesion has been identified such as dwarf mutants *an1* , *d1* , *d3* and *d5* . It also includes over 3200 mutants whose molecular lesion has neither been located within the genome nor molecularly characterized. These so called “phenotype-only” mutants are potentially a rich source from which to identify new alleles or genes acting in genetic pathways. Here, we evaluate 20 “phenotype-only’ stocks that have been visually scored as having the characteristic GA deficient, dwarf phenotype. The mutants were crossed to *an1* , *d1* , *d3* , and *d5* recessive dwarf mutants to determine allelism.

All but one of the 20 d* mutants were found to be allelic to *an1* , *d1* , *d3* or *d5* ( Figure 1 ). Four were allelic to *an1* , four to *d1,* eight to *d3* , and three to *d5* . Most of d* mutants presented the characteristic dwarf phenotype, *i.e.* rosette leaf structure, thick tassels with non-dehiscent anthers, and short internodes. The d*-teo mutants were atypical in this regard displaying a more moderate phenotype. Initially, the d*-teo seedlings and young plants displayed the compressed rosette phenotype common to dwarf mutants. However, around V10 stage the internodes above the ear elongated more than the internodes below the ear, unlike *d1* -6061 reference line, which remained compressed. The top ear of d*-teo mutants also developed sufficiently to enable pollination and kernel formation ( Figure 1 ). Despite the phenotypic differences between d*-teo and *d1* -6061, tests clearly demonstrated that these two mutants were alleles ( Figure 1 ). One potential explanation for the internode elongation above the ear in d*-teo is increased GA production later in the development of the mutant.

d*-ts1 was the only stock that failed to produce dwarf seedlings when crossed to *an1* , *d1* , *d3* , or *d5* . The stock showed clear segregation of dwarf seedlings from self-pollinated ears but failed to show segregation of dwarf seedlings in outcrosses to the reference heterozygous plants. This demonstrates that d*-ts1 is not an allele of the four dwarf tester loci, and this allele is encoded by yet another locus that either affects GA or another molecular pathway. One alternative explanation for a negative complementation test is that a homozygous normal plant was used for the outcross rather than an *an1* , *d1* , *d3* , or *d5* heterozygous plant. The experiment was designed such that all *an1* , *d1* , *d3* , and *d5* tester plants were either heterozygous or homozygous mutant. We did not confirm their genotype using genetic markers, so it is possible a contaminating homozygous normal plant was used in a cross. However, we detected no homozygous normal plants in the 41 outcrosses between the confirmed d* heterozygous plants and the allelic *d1* , *d3* or *d5* plants. This shows the homozygous normal contaminants were rare (or altogether absent), and unlikely to affect the interpretation of the allele tests when multiple outcrosses were conducted. Several allele tests were conducted by crossing homozygous d* plants to either heterozygous or homozygous reference mutants and resulted in higher frequency dwarf seedlings as expected.

## Methods

**Plant Materials**

All dwarf mutants used in this study are available from the Maize Genetics Cooperation Stock Center. The stock numbers for the four tester stocks were 116G *an1* , 302A *d1* -6061, 917FB *d3* -015-12, 214C *d5* . The stock numbers for the 20 unmapped dwarf* stocks were 4402K d*-N299B , 4404E d*-N629B, 4404O d*-N998B, 4405A d*-N1013B, 4405N d*-N1883, 4406C d*-N2248A, 4406F d*-N2252, 4406J d*-N2256, 4406L d*-N2278 , 4407I d*-N2506, 5503A d*-018-3, 5503C d*-136-220, 5503I d*-56-3037-23 , 5503K; d*-60-2447-8 , 5504L d*-gl11, 5505J d*-PI251656, 5505O d*-rosette, 5504K d*-Brawn254-258, 5505S d*-teo , and 5505T d*-ts1.

**Allele Test Design and Evaluation**

Allele test between d* mutants and *an1* , *d1* , *d3* , and *d5* testers were conducted at the University of Illinois Urbana-Champaign Crop Sciences Research and Education Center (South Farms) in 2023 and 2024 nurseries. For each d* mutant, one 4.5m row was planted using kernels selected from self-pollinated ears known to be segregating for the dwarf mutant plants. For the *d3* and *d5* reference alleles, 4.5m rows were planted using heterozygous kernels generated by outcrossing a homozygous mutant dwarf plant to a hybrid plant. Kernels from the *d1* reference allele were generated from a sibling cross pollination between a homozygous *d1/d1* plant and a heterozygous *d1/+* plant. The *d1* rows segregated 1:1 dwarf:normal plants. In most cases, allele tests were conducted by self-pollinating 4 normal (non-dwarf) plants from each d* line and outcrossing to heterozygous *an1* , *d1* , *d3* and *d5* reference allele plants. Two d* mutants shed sufficient pollen to allow for the mutant plant to be outcrossed to heterozygous and homozygous testers.

Allele test crosses were harvested, dried, shelled and 30 kernels from each self-pollinated ear and outcrossed ear were planted into sand bench rows. Seedlings were evaluated for two weeks after planting and scored for the number of dwarf and normal seedlings.
